# Effects of adult aging on reading filtered text: evidence from eye movements

**DOI:** 10.7717/peerj.63

**Published:** 2013-04-09

**Authors:** Kevin B. Paterson, Victoria A. McGowan, Timothy R. Jordan

**Affiliations:** College of Medicine, Biological Sciences and Psychology, University of Leicester, United Kingdom

**Keywords:** Human aging, Eye movements, Reading, Spatial frequencies

## Abstract

**Objectives.** Sensitivity to spatial frequencies changes with age and this may have profound effects on reading. But how the actual contributions to reading performance made by the spatial frequency content of text differs between young (18–30 years) and older (65+ years) adults remains to be fully determined. Accordingly, we manipulated the spatial frequency content of text and used eye movement measures to assess the effects on reading performance in both age groups.

**Method.** Sentences were displayed as normal or filtered to contain only very low, low, medium, high, or very high spatial frequencies. Reading time and eye movements were recorded as participants read each sentence.

**Results.** Both age groups showed good overall reading ability and high levels of comprehension. However, for young adults, normal performance was impaired only by low and very low spatial frequencies, whereas normal performance for older adults was impaired by all spatial frequencies but least of all by medium.

**Conclusion.** While both young and older adults read and comprehended well, reading ability was supported by different spatial frequencies in each age group. Thus, although spatial frequency sensitivity can change with age, adaptive responses to this change can help maintain reading performance in later life.

Normal reading relies on making a series of saccadic eye movements along lines of text, separated by periods of brief fixational pauses during which the eye is relatively stationary and visual information is acquired from the text (for reviews, see Rayner, 1998; Rayner, 2009). However, many studies show that older adults (aged 65+ years) often have greater difficulty in reading compared to young adults (typically aged 18–30; e.g., Kemper, Crow & Kemtes, 2004; Kliegl et al., 2004; Rayner, Castelhano & Yang, 2009; Rayner et al., 2006; see also Paterson, McGowan & Jordan, in press). Indeed, according to these studies, older adults often take longer to read, make more and longer fixational pauses, longer progressive saccades (forward eye movements in text) and more regressions (backwards eye movements in text) than young adults. This age-related difference in reading ability is widely attributed to sensory and cognitive decline associated with normal aging that may lead older adults to adopt a different reading strategy in order to compensate for their poorer processing of text (Rayner et al., 2006; Rayner, Castelhano & Yang, 2009). However, the nature of this decline, and how it affects older adults’ reading performance, has yet to be fully determined.

Of particular importance for the present research is that visual abilities change with normal aging, and that older adults often experience a range of subtle visual deficits that may affect their use of the spatial frequency content of words during reading (e.g., Akutsu et al., 1991; Elliott, Yang & Whitaker, 1995; for a recent review, see Owsley, 2011). It has long been established that the human visual system operates in the spatial frequency domain, supported by extensive psychophysical and anatomical evidence that pathways in the human visual system are sensitive to spatial frequencies associated with different scales of visual information (e.g., Blakemore & Campbell, 1969; Lovegrove et al., 1980; Robson, 1966). Accordingly, although words may appear to be composed only of letters, words are actually perceived as complex visual stimuli that contain a variety of spatial frequencies (e.g., Allen et al., 2009; Ginsburg, 1986; Patching & Jordan, 2005a; Patching & Jordan, 2005b). These range from lower spatial frequencies that provide coarse-scale visual information which may be useful for determining the overall layout of text, including the size, shape, and location of words on the page, to higher spatial frequencies that provide fine visual detail that may help to specify individual letters and letter features.

However, normal aging is associated with a progressive loss of visual sensitivity, particularly for higher spatial frequencies (e.g., Derefelt, Lennerstrand & Lundh, 1979; Elliott, 1987; Elliott, Yang & Whitaker, 1995; Higgins et al., 1988; Owsley, Sekuler & Siemsen, 1983; see Owsley, 2011), due to optical changes and changes in neural transmission that occur with increasing age. The precise effect of these changes on the reading abilities of older adults is unknown. But if this loss of sensitivity leads to a change in the functionality of various spatial frequencies during reading, young and older adults may differ in their use of the spatial frequency content of text, and this may have important consequences for understanding age differences in reading. Consequently, the aim of the present research was to investigate whether young and older adults show differences in the use of spatial frequencies during reading.

An approach taken previously to revealing the role of spatial frequencies in reading compared the reading performance obtained when text was shown normally with that when the spatial frequency content of text was filtered so that the text contained only certain spatial frequencies. The logic of this approach is that if readers require particular spatial frequencies to be present in text to read normally, normal reading performance will be unaffected when these spatial frequencies are present and impaired when they are absent. For instance, Legge et al. (1985) removed medium and higher spatial frequencies from text so that only lower spatial frequencies remained. This filtered text was then presented to several college-aged participants (aged 20–30 years) in a scrolling display in which lines of text moved from right to left so that participants could read each line aloud without moving their eyes. The results showed that reading rates for filtered text were largely unaffected relative to reading rates for unfiltered (normal) text, and this led Legge et al. (1985) to propose that only these lower spatial frequencies are required for reading. In a similar vein, Leat & Munger (1994) filtered sentences so that only certain spatial frequencies remained and found young adults read equally fluently when only low, medium, or high spatial frequencies were present, and this led Leat and Munger to argue that a broad range of spatial frequencies can support normal reading. Chung & Tjan (2009) filtered words so that they contained only certain spatial frequencies, and used a rapid serial visual presentation (RSVP) paradigm in which these words were presented sequentially at the same screen location and young adults read them aloud. The results showed that words displayed in a broad range of spatial frequencies were read equally quickly, and that reading rates were reduced when only very low or very high spatial frequencies were present, and this led Chung & Tjan (2009) to suggest that normal reading requires only certain spatial frequencies. Some support for this view is also provided by single-word recognition experiments showing that words are recognized most accurately when they contain only medium or relatively high or low spatial frequencies, and least accurately when they contain only very high or very low spatial frequencies (Patching & Jordan, 2005a; Patching & Jordan, 2005b).

Overall, these previous findings suggest that a wide range of spatial frequencies can support reading, and some studies even suggest that reading uses only a narrow subset of the normal spatial frequency content of text. However, much of this research has relied on measures of overall reading speed and recognition accuracy to assess reading performance without analyses of eye movement behavior, and some studies have intentionally employed techniques in which eye movements are not required. Studies that have assessed reading rates for text have also typically examined reading aloud, and considerable evidence shows that, compared to normal (silent) reading, reading aloud produces substantially different performance because readers articulate each word as it is encountered and fixations remain in the same place for longer (Lévy-Schoen, 1981; see also Rayner, 1998; Rayner, 2009). As a result, this research is not instructive about how the spatial frequency content of text supports processes of eye movement control during normal reading. Moreover, a wealth of research shows that eye movements are remarkably informative about cognitive processes that govern when and where to move the eyes during reading and can shed light on variation in reading behavior (Rayner, 1998; Rayner, 2009; see also Young et al., 2011), including differences in eye movement performance that occur as a result of normal aging.

More recent research has used eye-tracking to reveal effects of spatial frequencies on readers’ eye movements (Jordan, McGowan & Paterson, 2012; Paterson, McGowan & Jordan, 2012; Paterson, McGowan & Jordan, in press). In one such study, young adults read text that was displayed either as normal or filtered so that only certain spatial frequencies remained. The results showed that no single band of spatial frequencies supported normal eye movement behavior, but this behavior was closest to normal when text contained either medium or higher spatial frequencies. Moreover, other research has manipulated spatial frequencies only at locations away from the reader’s point of gaze but suggests there are age differences in the use of spatial frequencies (Paterson, McGowan & Jordan, in press). However, this research was not concerned with effects of filtering the spatial frequency of text both at locations within central vision and more peripheral locations, and so it not fully informative about adult age differences in the function of various spatial frequencies during reading.

Accordingly, to gain a fuller understanding of the effects of normal aging on the role of spatial frequencies during reading, the present research assessed the reading times and eye movement behavior of young and older adults when reading sentences displayed as normal or filtered to contain only very low, low, medium, high, or very high spatial frequencies (see Fig. 1). If no single range of spatial frequencies supports normal reading, reading times should be longer and normal patterns of eye movements should be disrupted when text is filtered compared to when text is displayed normally. But more importantly, the functional relevance of different spatial frequencies to normal reading should become apparent for each age group. In particular, if young adults rely mostly on medium, high and very high spatial frequencies to read (Paterson, McGowan & Jordan, 2012), these frequencies should disrupt normal reading the least. By comparison, if older adults find higher spatial frequencies less beneficial for reading (due to age-related changes in visual sensitivity), these higher frequencies should not produce the same advantage for this age group.

**Figure 1:**
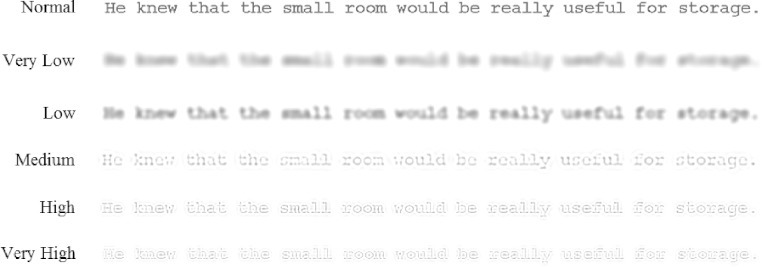
Examples of a sentence shown in each display condition.

## Method

### Participants

Participants were 12 young adults (*M* = 21 years, range = 18–30 years) and 12 older adults (*M* = 73 years, range = 65–79 years) from the University of Leicester and the local community. All participants were native speakers of English and both age groups had similar educational backgrounds. Screening established that participants did not suffer from eye disease or disorders, or visual or reading impairment (e.g., dyslexia). Participants’ visual abilities were assessed using Bailey–Lovie (Bailey & Lovie, 1976), ETDRS (Ferris & Bailey, 1996), and Pelli-Robson (Pelli, Robson & Wilkins, 1988) tests. Assessments confirmed that participants were properly refracted at the screen distance used in the experiment. Older adults showed typical lower acuity than young adults (young adults, *M* = 20/18, older adults, *M* = 20/25, where acuity is reported in Snellen values). Older adults also showed lower sensitivity to higher spatial frequencies than young adults (log contrast sensitivity: young adults, *M* = 1.81, older adults, *M* = 1.76), which again is typical for these age groups (e.g., see Owsley, 2011). Ethical approval for this research was obtained from the School of Psychology Ethics Committee at the University of Leicester (Ref. kbp3-b004). Informed written consent was obtained from all participants.

### Stimuli and design

Stimuli consisted of 120 sentences. Each sentence was shown in 1 of 6 display conditions, shown either entirely as normal (unfiltered) or with the spatial frequency content of the text filtered so that only one of 5 bands of restricted spatial frequencies were present, ranging from very low to very high (see Fig. 1). This filtered text was created using MATLAB to digitally filter each sentence into 5 different, 1-octave wide bands of spatial frequencies with peak frequencies of 2.2, 3.5, 4.9, 6.7, 11.1, and 13.7 cycles per degree (cpd) and low-pass and high-pass cut-off frequencies of 1.65–3.3, 2.6–5.2, 5.0–10.0, 8.3–16.6, and 10.3–20.6 cpd (for further details, see Patching & Jordan, 2005a; Patching & Jordan, 2005b). This was achieved by point-wise multiplication in the frequency domain using fourth-order high- and low-pass Butterworth filters. These filters provide a mathematically tractable filter shape that avoids the problems of ringing associated with other filter shapes with a sharp cut-off. The 5 bands of spatial frequencies produced were termed very low, low, medium, high, and very high.

All 120 sentences were randomized and sampled using a Latin square so that each participant saw 20 sentences shown in each of the 6 display conditions. This ensured that all sentences were shown equally often in each condition in the experiment but prevented repetition of any sentence for any participant. Sentences were shown to each participant in a randomized order across two sessions. An additional 12 sentences (2 per condition) were presented as practice items, 6 at the beginning of each session. Indications of the displays used in the experiment are shown in Fig. 1.

### Apparatus

Eye movements were recorded using an Eyelink 2K tower-mounted eye-tracker with chin and forehead rest. This eye-tracker has a spatial resolution of .01° and the position of each participant’s right eye was sampled at 1000 Hz using corneal reflection and pupil tracking. Sentences were displayed on a 19 inch monitor and a 4-letter word subtended approximately 1° (i.e., normal size for reading; Rayner & Pollatsek, 1989).

### Procedure

At the beginning of the experiment, participants were informed that sentences might be difficult to read, and that they should always attempt to read normally, and for comprehension. The eye-tracker was then calibrated. At the start of each trial, a fixation square (equal in size to 1 character space) was presented in the center left of the screen. Once the participant fixated this location accurately, a sentence was presented, with the first letter of the sentence replacing the square. Participants were instructed to press a response key once they finished reading each sentence. Each sentence was then replaced by a comprehension question about the content of the sentence (e.g., “Was the room useful for storage?”) to which each participant responded. Calibration was checked between trials and the eye-tracker was recalibrated as necessary.

## Results

A range of eye movement measures was computed. These were reading times, average fixation duration (the average length of fixational pauses during reading), number of fixations (the number of these fixational pauses), length of progressive saccades (the length, in characters, of forward eye movements in the text), and number of regressions (the number of backward movements in the text). Comprehension accuracy for questions that followed each sentence was also recorded (see Table 1). Effects of age group (young adults, older adults) and display condition (normal plus 5 types of spatial frequency filter) on these measures were analyzed using a 2 × 6 mixed design Analysis of Variance (ANOVA) computing error variance over participants (*F*_1_) and sentences (*F*_2_), and using the Greenhouse-Geisser correction where appropriate. We report partial eta-squared (${\eta }_{p}^{2}$) as a measure of effect size. Pairwise comparisons were performed using a Tukey test (*p* < .05 for significant effects).

### Comprehension accuracy

There was no main effect of age group (young adults = 90%, older adults = 86%), *F* *s* < 2. However, comprehension accuracy varied across the display conditions, (normal = 95%, very low = 67%, low = 92%, medium = 95%, high = 91%, very high = 87%), *F*_1_(5,110) = 31.25, *p* < .001, ${\eta }_{p}^{2}=.59$, and *F*_2_(5,595) = 48.62, *p* < .001, ${\eta }_{p}^{2}=.29$, and there was an interaction between these factors, *F*_1_(5,110) = 5.14, *p* < .001, ${\eta }_{p}^{2}=.19$, and *F*_2_(5,595) = 10.12, *p* < .001, ${\eta }_{p}^{2}=.08$. This interaction was due to significantly lower accuracy (compared to normal sentence displays) for the very low spatial frequency displays for both age groups, and for the very high spatial frequency displays for the older adults only. There was no significant age difference in comprehension accuracy for text displayed normally, and no other differences were significant. Thus, although readers comprehended text normally when it was displayed in a broad range of spatial frequencies, normal comprehension was impaired for both young and older adults when text contained only very low spatial frequencies and for older adults when text contained only very high spatial frequencies.

### Reading time

**Figure 2:**
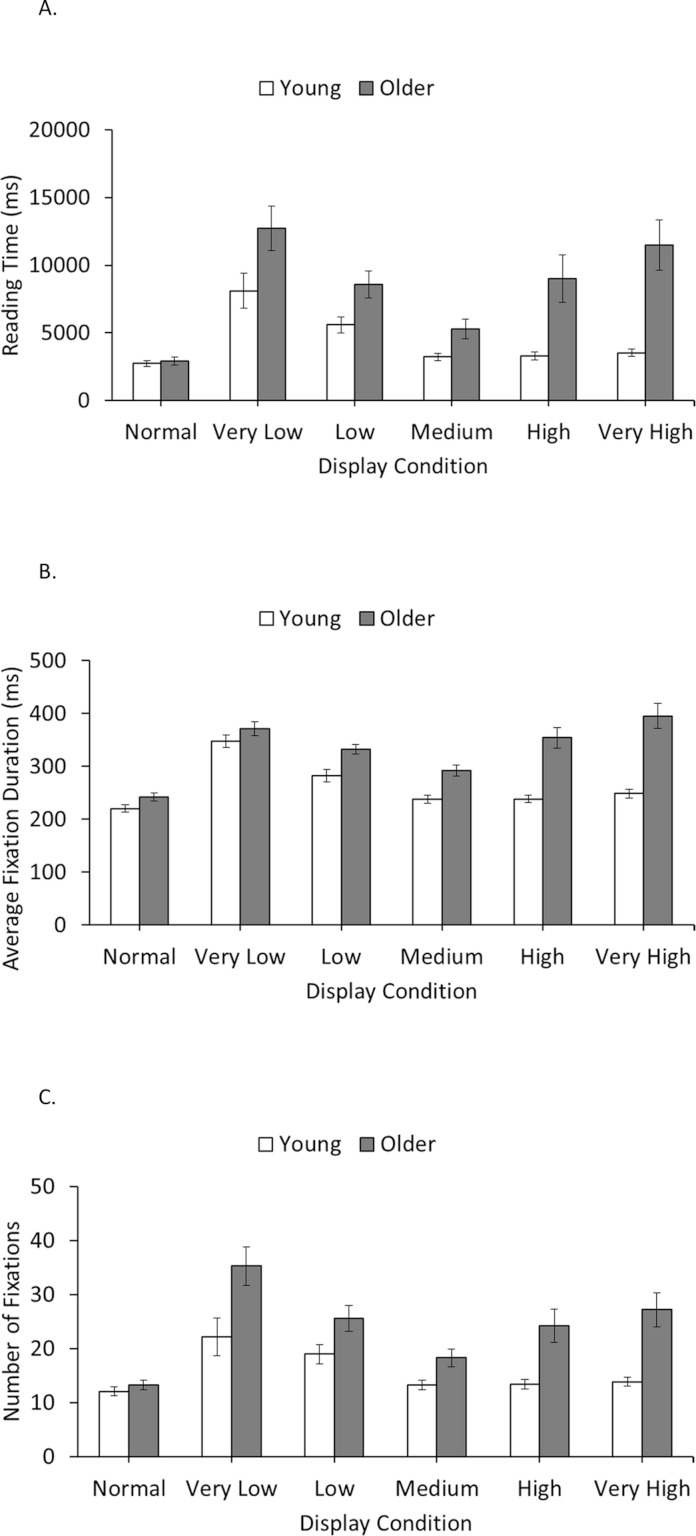
Reading times (A), average fixation durations (B), and number of fixations (C) for young and older adults in each display condition. Error bars represent the Standard Error.

Mean reading times are shown in Fig. 2A. There were main effects of age group (young adults = 4413 ms, older adults = 8504 ms), *F*_1_(1, 22) = 13.44, *p* < .01, ${\eta }_{p}^{2}=.38$, and *F*_2_(1, 119) = 2369.10, *p* < .001, ${\eta }_{p}^{2}=.95$, and display condition (normal = 2839 ms, very low = 10924 ms, low = 7078 ms, medium = 4257 ms, high = 6143 ms, very high = 7508 ms), *F*_1_(5,110) = 27.62, *p* < .001, ${\eta }_{p}^{2}=.56$, and *F*_2_(5,595) = 99.67, *p* < .001, ${\eta }_{p}^{2}=.46$, and an interaction between these factors, *F*_1_(5,110) = 7.09, *p* < .001, ${\eta }_{p}^{2}=.24$, and *F*_2_(5,595) = 27.55, *p* < .001, ${\eta }_{p}^{2}=.19$. For young adults, reading times were significantly longer (compared to normal) for low and very low spatial frequencies but differed little from normal for medium, high, or very high spatial frequencies. For older adults, reading times were significantly longer (compared to normal) for all spatial frequencies. These were closest to normal for medium spatial frequencies, but significantly longer for low, high, and very high spatial frequencies, and significantly longer still for very low spatial frequencies. Further analyses showed that reading times did not differ significantly between young and older adults for text shown normally, but were significantly longer for older adults for all other display conditions.

### Average fixation duration

Mean average fixation durations are shown in Fig. 2B. There were main effects of age group (young adults = 262 ms, older adults = 329 ms), *F*_1_(1, 22) = 22.64, *p* < .001, ${\eta }_{p}^{2}=.35$, and *F*_2_(1, 119) = 1802.07, *p* < .001, ${\eta }_{p}^{2}=.94$, and display condition (normal = 231 ms, very low = 359 ms, low = 307 ms, medium = 264 ms, high = 294 ms, very high = 319 ms), *F*_1_(5,110) = 21.96, *p* < .001, ${\eta }_{p}^{2}=.50$, and *F*_2_(5,595) = 395.36, *p* < .001, ${\eta }_{p}^{2}=.77$, and an interaction between these factors, *F*_1_(5,110) = 18.39, *p* < .001, ${\eta }_{p}^{2}=.46$, and *F*_2_(5,595) = 79.56, *p* < .001, ${\eta }_{p}^{2}=.40$. For young adults, average fixation durations were significantly longer (compared to normal) for low and very low spatial frequencies, but differed little from normal for medium, high, or very high spatial frequencies. For older adults, average fixation durations were significantly longer (compared to normal) for all spatial frequencies but were closest to normal for medium spatial frequencies, significantly longer for low and high spatial frequencies, and significantly longer still for very low and very high spatial frequencies. Further analyses showed that average fixation durations did not differ significantly between young and older adults for very low spatial frequencies, but were significantly longer for older adults for text shown normally and for all other filter conditions.

### Number of fixations

Mean numbers of fixations are shown in Fig. 2C. There were main effects of age group (young adults = 15.6, older adults = 23.0), *F*_1_(1, 22) = 12.58, *p* < .01, ${\eta }_{p}^{2}=.28$, and *F*_2_(1, 119) = 1318.88, *p* < .001, ${\eta }_{p}^{2}=.92$, and display condition (normal = 12.7, very low = 28.8, low = 22.3, medium = 15.8, high = 18.8, very high = 20.5), *F*_1_(5,110) = 22.52, *p* < .001, ${\eta }_{p}^{2}=.45$, and *F*_2_(5,595) = 53.34, *p* < .001, ${\eta }_{p}^{2}=.31$, and a significant interaction between these factors, *F*_1_(5,110) = 4.33, *p* < .05, ${\eta }_{p}^{2}=.16$, and *F*_2_(5,595) = 24.04, *p* < .001, ${\eta }_{p}^{2}=.17$. Young adults made more fixations compared to normal sentence displays for low and very low spatial frequencies, but fixation counts for medium, high, and very high spatial frequencies were not significantly different from normal displays. Older adults made more fixations (compared to normal) for all filter conditions. The older adults’ number of fixations was closest to normal for medium spatial frequencies, significantly higher for high, very high and very low spatial frequencies, and significantly higher still for very low spatial frequencies. Further analyses showed that number of fixations did not differ significantly between young and older adults for normal text, but was significantly higher for older adults for all filter conditions.

### Progressive saccade length

Mean progressive saccade lengths are shown in Table 1. There was a main effect of age group (marginally significant for the *F*_1_ analysis; young adults = 8.1 characters, older adults = 9.2 characters), *F*_1_(1, 22) = 3.99, *p* < .06, ${\eta }_{p}^{2}=.15$, and *F*_2_(1, 119) = 32.32, *p* < .001, ${\eta }_{p}^{2}=.21$, a main effect of display condition (normal =10.3 characters, very low = 8.2 characters, low = 8.2 characters, medium = 9.0 characters, high = 8.3 characters, very high = 8.4 characters), *F*_1_(5,110) = 18.10, *p* < .001, ${\eta }_{p}^{2}=.45$, and *F*_2_(5,595) = 64.71, *p* < .001, ${\eta }_{p}^{2}=.35$, and an interaction between these factors, *F*_1_(5,110) = 2.84, *p* < .05, ${\eta }_{p}^{2}=.11$, and *F*_2_(5,595) = 4.79, *p* < .01, ${\eta }_{p}^{2}=.04$. Both young and older adults made shorter progressive saccades (compared to normal) for all filter conditions. For young adults, progressive saccade length was equally shorter than normal for all filter conditions, but for older adults, progressive saccade length was closest to normal for medium and low spatial frequencies and significantly longer for all other spatial frequencies. Further analyses showed that saccade length was significantly longer for older adults than young adults for normal text and low spatial frequencies, but did not differ significantly between age group for any other display condition.

### Number of regressions

Mean numbers of regressions are shown in Table 1. There were main effects of age group (young adults = 1.7, older adults = 4.6), *F*_1_(1, 22) = 16.30, *p* < .05, ${\eta }_{p}^{2}=.43$, and *F*_2_(1, 119) = 885.37, *p* < .001, ${\eta }_{p}^{2}=.88$, and display condition (normal = 2.2, very low = 4.5, low = 3.6, medium = 2.5, high = 2.8, very high = 3.2) *F*_1_(5,110) = 17.55, *p* < .001, ${\eta }_{p}^{2}=.44$, and *F*_2_(5,595) = 79.36, *p* < .001, ${\eta }_{p}^{2}=.40$, and an interaction between these factors, *F*_1_(5,110) = 4.95, *p* < .001, ${\eta }_{p}^{2}=.18$, and *F*_2_(5,595) = 40.08, *p* < .001, ${\eta }_{p}^{2}=.25$. Young adults made more regressions than normal for only very low spatial frequencies, but older adults made more regressions than normal for all filter conditions. For older adults, the number of regressions was closest to normal for medium spatial frequencies, significantly greater for high spatial frequencies, significantly greater still for low and very high spatial frequencies, and significantly greatest for very low spatial frequencies. Further analyses showed that older adults made significantly more regressions than young adults for normal text and for all filter conditions.

**Table 1:** Comprehension accuracy, progressive saccade length, and number of regressions by young and older adults in each display condition.

	Display condition											
	Normal		Very low		Low		Medium		High		Very high	
	Young	Older	Young	Older	Young	Older	Young	Older	Young	Older	Young	Older
Comprehension accuracy (%)	93 (1.7)	97 (1.0)	66 (3.2)	68 (2.8)	95 (2.1)	88 (4.1)	94 (1.9)	95 (1.2)	94 (1.9)	89 (3.0)	97 (1.1)	76 (6.8)
Progressive saccade length (Characters)	9.1 (0.4)	11.4 (0.4)	7.9 (0.5)	8.5 (0.3)	7.6 (0.3)	8.8 (0.4)	8.4 (0.4)	9.6 (0.4)	8.0 (0.4)	8.4 (0.4)	7.6 (0.3)	9.2 (1.0)
Number of regressions	2.4 (0.7)	4.4 (0.6)	5.3 (1.5)	12.4 (1.7)	4.6 (0.9)	9.0 (1.1)	2.8 (0.5)	5.9 (0.7)	2.5 (0.5)	7.9 (1.3)	2.6 (0.4)	9.3 (1.6)

## Discussion

The major indication provided by this study is that important adult age differences exist in the functionality of spatial frequencies during reading. This difference was observed particularly clearly in reading times, average fixation durations, and the number of fixations made while reading, but was also observed in other eye movement measures (progressive saccade length, number of regressions) and in comprehension accuracy, indicating that young and older readers used different spatial frequencies in text to support normal processes of comprehension.

The findings for young adults showed that reading times, average fixation durations, and number of fixations differed little from normal for medium, high, and very high spatial frequencies, but were increased for low and very low. This advantage for medium and higher spatial frequencies resembles that observed previously for young adults (Paterson, McGowan & Jordan, 2012) and is consistent with research showing that a broad range of spatial frequencies can support normal reading (Chung & Tjan, 2009; Jordan, McGowan & Paterson, 2012; Leat & Munger, 1994; Legge et al., 1985; Patching & Jordan, 2005a; Patching & Jordan, 2005b; Paterson, McGowan & Jordan, 2012; Paterson, McGowan & Jordan, in press). Thus, the indication is that young adults do not require the full complement of spatial frequencies ordinarily present in text for these aspects of reading behavior to be normal. Instead, this age group appears to function best using spatial frequencies that provide fine visual detail that may help identify individual letters and letter features as well as determine the location and boundaries of words on the page. This was not simply an effect on reading behaviour, however, but also affected comprehension, and while a broad range of spatial frequency content, from low to very high, supported normal levels of comprehension (>90%), young adults found that reading for comprehension was impaired (only 66% accurate) for very low spatial frequencies.

Compared to young adults, older adults showed different patterns of performance for filtered text. In particular, older adults had longer reading times and fixation durations, and made more fixations for all spatial frequencies compared to normal text, and so experienced greater reading difficulty than young adult readers when text lacked its normal full complement of spatial frequencies. In addition, older adults’ reading times, fixation durations, and number of fixations were close to normal only for medium spatial frequencies and much longer than normal for both lower and higher spatial frequencies. Thus, the benefits of higher spatial frequencies observed for young adults were not apparent for older readers. Moreover this age-related difference did not affect only reading behavior, as levels of comprehension also indicated a more limited range of usable spatial frequencies for older adults and showed that performance was much lower than normal for both very high and very low spatial frequencies. Although memory processes also typically decline with older age (e.g., Craik, 1994), there was no indication that these processes affected older adults’ comprehension accuracy when text was displayed as normal, and this suggests that the effects observed for filtered text were unlikely to be related to memory processes.

Other eye movement measures provided additional evidence for age-related changes in the use of spatial frequencies. In particular, both young and older adults made shorter progressive saccades and more regressions for all spatial frequencies compared to normal text, consistent with both age groups having greater difficulty in reading and so adopting a more cautious oculomotor strategy in which they make shorter forward saccades than normal when text lacks its full complement of spatial frequencies. However, whereas saccade length and regression rates were affected equally by all spatial frequencies for young adults, older adults’ progressive saccade length was affected least by medium and low spatial frequencies, and their regression rates were affected least by medium spatial frequencies. Thus, a much narrower range of spatial frequencies appear to be effective in supporting normal patterns of eye guidance in older adult readers.

The overall indication, therefore, is that older adults generally require a broader range of spatial frequencies for reading to be normal and do not display the benefits for higher spatial frequencies observed for young adults. These differences in the effectiveness of spatial frequencies across age groups suggest that the contribution made by any single band of spatial frequencies was too weak to support normal reading by older adults and that higher spatial frequencies made the weakest contribution. Indeed, the reduced effectiveness observed for older adults is in line with a general age-related decline in sensitivity to spatial frequencies that is widely-observed for older adults and is most pronounced for higher spatial frequencies associated with perception of fine visual detail (e.g., Derefelt, Lennerstrand & Lundh, 1979; Elliott, 1987; Higgins et al., 1988; Owsley, Sekuler & Siemsen, 1983; see Owsley, 2011). Thus, the present findings suggest that reading is not immune to changes in visual sensitivity that occur as adults grow older, and that age-related changes in spatial-frequency sensitivity have profound effects on eye-guidance as adults reach older age.

However, despite clear age differences in the influence of spatial frequencies, both age groups produced near identical levels of reading times (and fixation counts) when text was displayed normally, although older adults made longer gaze durations, longer progressive saccades and more regressions. These findings resonate well with previous studies (Kemper, Crow & Kemtes, 2004; Kliegl et al., 2004; Rayner et al., 2006; Rayner, Castelhano & Yang, 2009) and with the findings reported by Paterson, McGowan & Jordan (in press) who also found no reading time difference between young and older adults for text shown normally but age-related differences in eye movement behaviour when text was filtered at locations away from gaze. The similarity in reading speeds observed for young and older adults in the present study, and by Paterson, McGowan & Jordan (in press), may reflect the care with which participants’ visual abilities were screened in both these studies in order to avoid misleading influences of clinical impairments. Indeed, the careful screening of visual abilities is of particular concern for research on older adults’ reading as clinical impairments are common in this population and can have profound effects on reading performance but often go unnoticed until accurate assessments are made (for a recent review, see McGowan, Paterson & Jordan, 2013).

The similarity in reading speeds for normal text and the high rates of comprehension accuracy observed for young and older adults also indicate that changes in sensitivity to various spatial frequencies with normal aging do not necessarily produce a catastrophic decline in reading ability. Instead, our findings suggest that, as readers get older, an adaptive shift in the use of spatial frequencies may develop so that information that is more visible becomes the most important for reading. Although adults show a general loss of visual sensitivity, this is greatest for higher spatial frequencies. As a result, older readers may at least partially compensate for this greater loss of visual sensitivity for higher spatial frequencies by relying more heavily on coarse-scale cues to word identities (including cues provided by supra-letter features in words, such as length and shape) when reading (and for other evidence of the importance of word boundaries for eye guidance, see Pollatsek & Rayner, 1982; Paterson & Jordan, 2010; Rayner, Fischer & Pollatsek, 1998). It may also be the case that older adults offset their loss of sensitivity to fine visual detail by greater use of contextual information, although the extent to which older adults benefit more from contextual cues during reading is controversial (e.g., Stine-Morrow, Miller & Nevin, 1999; Federmeier & Kutas, 2005; Federmeier, Kutas & Schul, 2010), and eye movement research suggests that older adults do not benefit more than young adults from the contextual predictability of words (Kliegl et al., 2004; Rayner et al., 2006). Indeed, as greater reliance on coarse-scale cues or contextual predictability may not always establish the identity of words during reading, this may help explain why older adults often appear to employ a “riskier” strategy when reading, where they guess the identities of words more frequently than young adults, and so are likely to make longer saccades (typically due to increased skipping of words) and to make more regressions to re-inspect words that have been misidentified (e.g., Rayner et al., 2006; Rayner, Castelhano & Yang, 2009). The longer progressive saccades and increased regressions for older adults when reading normal text in the present experiment are consistent with this account. Indeed, this general approach resonates with the widely-held view that greater experience of language and reading that accrues from habitual engagement with text throughout adulthood may buffer against effects of sensory decline in later years (see, e.g., Stine-Morrow, Miller & Herzog, 2006).

In sum, the performance of both age groups in the present study supports the view that reading involves information from a range of spatial scales and that a wide range of spatial frequencies contributes to reading performance (e.g., Allen & Madden, 1991; Allen et al., 2009; Boden & Giaschi, 2009; Jordan, 1990; Jordan, 1995; Jordan et al., 2003; Leat & Munger, 1994; Legge et al., 1985; Patching & Jordan, 2005a; Patching & Jordan, 2005b). Moreover, our findings suggest that the spatial frequencies that are most effective for reading change substantially during the adult lifespan and these changes produce important influences on eye-guidance. However, our findings also suggest that although the influence of different spatial frequencies may change, adaptive responses to these changes help older adults maintain reading performance well into later life.
